# Métastases pleuropulmonaires révélant un mélanome malin de la conjonctive chez un sujet jeune

**DOI:** 10.11604/pamj.2016.23.98.7246

**Published:** 2016-03-15

**Authors:** Hanane El Ouazzani, Hicham Janah, Sabah Alami El Machichi, Leila Achachi, Mohamed Taoufiq El Fassy Fihry, Mohamed El Ftouh

**Affiliations:** 1 Service de pneumologie, Hôpital Ibn Sina, Faculté de médecine et de pharmacie Rabat, Maroc

**Keywords:** Mélanome, métastases, conjonctive, Melanoma, metastasis, conjunctiva

## Abstract

Le mélanome de la conjonctive est une tumeur rare avec une incidence de 0,03 à 0,08 pour 100000 dans la population blanche. Le mélanome malin métastatique constitue environ 5% de toutes les tumeurs malignes secondaires du poumon. Nous rapportons un cas de métastase pleurale et pulmonaire d'un mélanome conjonctival de découverte fortuite chez un sujet jeune.

## Abstract (en)

Melanoma of the conjunctiva is a rare tumor with incidence from 0,03 to 0,08 cases per 100000 white population. Metastatic malignant melanoma represents approximately 5% of all secondary malignant lung tumors. We report the unexpected discovery of a pulmonary and pleural metastasis from a conjunctival melanoma in a young subject.

## Introduction

Le mélanome de la conjonctive est une tumeur oculaire peu fréquente. Il s'agit d'une tumeur de mauvais pronostic en raison de son caractère récidivant et de son pouvoir métastatique [1]. La précocité de survenue des métastases est rare et les pleurésies sont une présentation peu fréquente [2]. Nous rapportons un cas de métastase pleurale et pulmonaire d'un mélanome conjonctival de découverte fortuite chez un sujet jeune.

## Patient et observation

**Figure 1:**
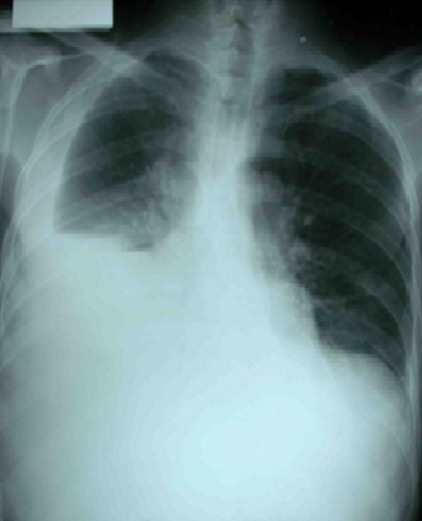
Radiographie thoracique montrant une opacité de tonalité hydrique basale droite et des images nodulaire parenchymateuse

**Figure 2:**
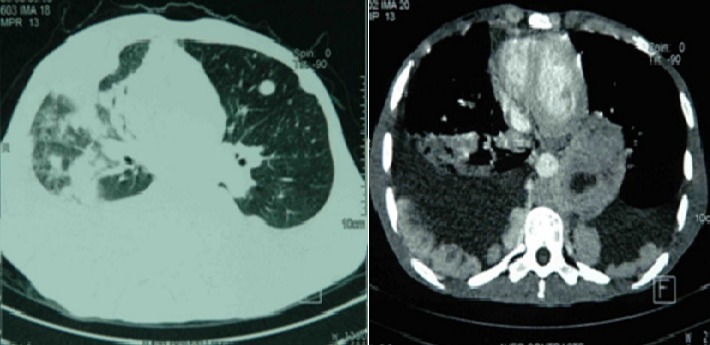
TDM thoracique a montré une pleurésie bilatérale avec des masses pleurales et des nodules parenchymateux pulmonaire bilatéraux

**Figure 3:**
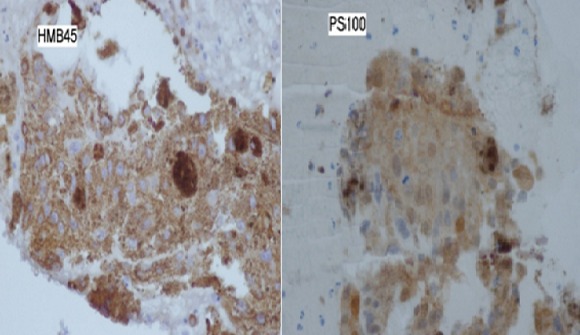
L’étude immuno histochimique montre un immunomarquage positif par les anticorps anti HMB 45 et anti protéine S100

**Figure 4:**
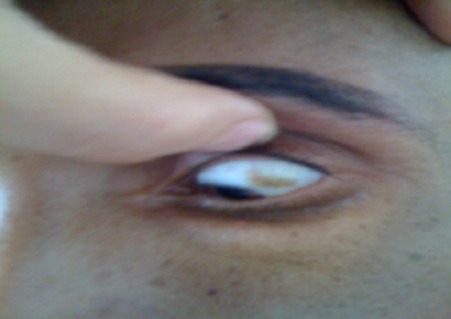
Mélanome malin de la conjonctive

Patient âgé de 27 ans, qui présentait depuis 2 mois une douleur thoracique droite à type de point de coté associée à une toux sèche et une dyspnée d'effort évoluant dans un contexte d'altération de l’état général avec un amaigrissement important chiffré à 8 kg en 2 mois. L'examen physique a objectivé un syndrome d’épanchement liquidien droit. La radiographie thoracique a montré une opacité de tonalité hydrique basale droite et des images nodulaires parenchymateuse (Figure 1). Le scanner thoracique a révélé une pleurésie bilatérale avec des masses pleurales et des nodules parenchymateux pulmonaires bilatéraux (Figure 2). La ponction pleurale a retiré un liquide sérohématique et l’étude anatomopathologique des biopsies pleurales réalisées a montré la présence d'une nappe cellulaire diffuse faite d’éléments de grande taille pourvus d'un cytoplasme abondant acidophile et chargé d'un pigment brunâtre. L’étude immuno histochimique a confirmé le diagnostic de métastases pleurales d'un mélanome malin avec un immunomarquage positif pour les anticorps anti HMB 45 et anti proteine S100 (Figure 3). L'examen cutané muqueux à la recherche du mélanome primitif n'a pas trouvé de lésions cutanées mais une lésion brunâtre au niveau de la conjonctive que le patient n'avait jamais remarquée (Figure 4). L'examen ophtalmologique confirme une localisation conjonctivale d'un mélanome malin. Vu l'altération importante de l’état général, la chimiothérapie a été récusé par les oncologues et on a réalisé une thoracoscopie avec talcage dans un but palliatif devant la récidive rapide de la pleurésie.

## Discussion

Le mélanome malin de la conjonctive est une néoplasie rare, puisqu'il ne représente que 2% des tumeurs malignes oculaires [3] et seulement 1,6% des mélanomes d'origine non cutanée [4]. Son incidence dans la population caucasienne étant estimée à 0,03-0,08 pour 100 000 habitants [5]. Le risque de métastase à distance est estimé à 26% à 10 ans et il ne semble pas exister de localisation métastatique préférentielle [4]. Desjardins et al ont observé dans une série rétrospective de 56 patients traités pour mélanome malin de la conjonctive, 17 cas de métastase (30%) et il s′agissait le plus souvent d′adénopathie locorégionale (23,5%) ou de métastase cérébrale (41%) [3]. Les métastases thoraciques du mélanome malin représentent approximativement 5% de toutes les métastases secondaires de poumon [6]. Peu de cas de métastase pleurale d'un mélanome cutaneomuqueux sont rapportées dans la littérature. Chen et al ont trouvé dans une série de 130 cas de métastase thoracique de mélanome malin, 2% seulement qui avaient une pleurésie métastatique [7]. La manifestation précoce des métastases du mélanome conjonctivale est rare. Dans la série de Desjardins et al, un cas seulement avait des métastases au moment du diagnostic [3]. En général, les métastase se manifestent tardivement au cours de la maladie quelques années après le diagnostic initial [8].

## Conclusion

Le mélanome malin de la conjonctive est une néoplasie rare et les métastases thoraciques de cette néoplasie sont encore plus rares. Face au peu de solutions thérapeutiques efficaces contre les métastases, il est important d'identifier très tôt les patients à risque pour leur proposer un traitement adjuvant, à la suite du traitement local.
